# Morphological and molecular characterization of *Cosmocercoides amapari* n. sp. (Nematoda: Cosmocercidae), parasitic in hylid frogs from the Brazilian Amazon

**DOI:** 10.1017/S0031182022001767

**Published:** 2023-03

**Authors:** Gabriel Lima Rebêlo, Ana Nunes Santos, Lorena Freitas Souza Tavares-Costa, Marcos Roberto Dias-Souza, Maria Isabel Müller, Ronald Ferreira Jesus, Carlos Eduardo Costa-Campos, Jeannie Nascimento dos Santos, Francisco Tiago de Vasconcelos Melo

**Affiliations:** 1 Laboratory of Cellular Biology and Helminthology ‘Profa. Dra. Reinalda Marisa Lanfredi’, Institute of Biological Sciences, Federal University of Pará (UFPA), Av. Augusto Correa 01, Guamá, Belém, Pará 66075-110, Brazil; 2 Department of Ecology and Evolutionary Biology, Federal University of São Paulo (Unifesp), Rua Professor Arthur Riedel, 275, Jardim Eldorado, Diadema, São Paulo 09972-270, Brazil; 3 Laboratory of Herpetology, Department of Biological and Health Sciences, Federal University of Amapá (UNIFAP), Rod. Josmar Chaves Pinto, km 02, Jardim Marco Zero, Macapá, Amapá 68903-419, Brazil

**Keywords:** Amapá, amphibians, integrative taxonomy, Neotropical, parasite

## Abstract

*Cosmocercoides* Wilkie, 1930 are gastrointestinal parasites commonly found in amphibians and reptiles, with 4 species reported from the Neotropical region. In the present study, a new species of *Cosmocercoides*, namely *Cosmocercoides amapari* n. sp. is described using integrated approaches such as light and scanning microscopy, and DNA sequencing of the mitochondrial cytochrome c oxidase subunit 1 gene. The specimens were collected from the large intestine of 3 species of hylid frogs in Amapá, Brazil. The new species can be distinguished from its congeners by morphological traits, including the pattern of caudal papillae, absence of the gubernaculum, 2 poorly sclerotized spicules, presence of lateral alae and somatic papillae along the body. In addition, our molecular analyses and phylogenetic reconstructions strongly support the status of the new taxon, which clustered poorly with a large clade of *Cosmocerca* spp. *Cosmocercoides amapari* n. sp. is the 29th species of the genus, the 5th from the Neotropical region, the third reported in Brazil, the second described from the Amazon region and the first belonging to the Neotropical region with molecular data.

## Graphical abstract

**Figure:**
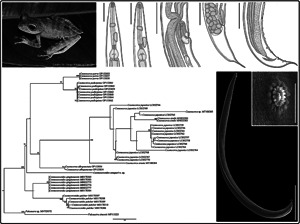

## Introduction

*Cosmocercoides* Wilkie, 1930 are gastrointestinal parasites commonly found in amphibians and reptiles, and occasionally in terrestrial snails and slugs. These nematodes are characterized mainly by the rosette papillae on the male caudal region (Chen *et al*., 2018*a*; Liu *et al*., 2019; Dos Anjos *et al*., 2021). The species have monoxenic life cycle and adult females release eggs in host feces that develop into first-stage larvae in the environment. The larvae moult twice and become an infective third-stage larva that penetrates through the skin of a new host (Anderson, 2000).

Currently, there are approximately 28 nominal species of *Cosmocercoides* distributed worldwide. Of those, only 4 species have been reported from the Neotropical region, including *Cosmocercoides lilloi* Ramallo *et al*., 2007 from *Rhinella arenarum* (Hensel, 1867); *Cosmocercoides latrans* Draghi *et al*., 2020 from *Leptodactylus luctator* (Hudson, 1892), both from Argentina; *Cosmocercoides sauria* Ávila *et al*., 2010 from lizard *Iphisa elegans* (Gray, 1851) and *Cosmocercoides meridionalis* Anjos *et al*., 2021 from *Boana geographica* (Spix, 1824), *Boana boans* (Linnaeus, 1758), *Dryaderces* cf. *inframaculata* (Boulenger, 1882), *Osteocephalus taurinus* (Steindachner, 1862) and *Phyllomedusa camba* (De la Riva, 1999), both from Brazil (Ramallo *et al*., 2007; Ávila *et al*., 2010; Draghi *et al*., 2020; Dos Anjos *et al*., 2021).

Until now, molecular data available for *Cosmocercoides* spp. are very scarce, and only *Cosmocercoides tonkinensis* Tran, Sato and Luc, 2015; *Cosmocercoides qingtianensis* Chen *et al*., 2018; *Cosmocercoides wuyiensis* Liu *et al*., 2019; *Cosmocercoides pulcher* Wilkie, 1930 from Oriental region and *Cosmocercoides dukae* Holl, 1928 from Nearctic region were studied using molecular tools (Chen *et al*., 2018*a*, 2020). Thus, we used an integrative approach, including light and scanning electron microscopy and molecular analysis, to describe and characterize a new species of *Cosmocercoides* and determine the phylogenetic position of this species.

## Materials and methods

### Host collection and morphological study of parasites

We carried out a parasitological survey in the Municipal Natural Park of ‘Cancão’, located in the municipality of Serra do Navio, Amapá, Brazil (0°54′8.68″N, 52°0′19.62″W), from 2015 to 2019. We collected 28 specimens of *B. boans*, 27 of *Boana dentei* (Bokermann, 1967) and 51 of *Boana multifasciata* (Günther, 1859) through an active search (permission number SISBIO: no. 48102-2/IBAMA/ICMBio).

The hosts were anaesthetized and euthanized with ketamine 2%, measured, weighed and necropsied for helminth search. All internal organs were placed in Petri dishes with saline solution (NaCl 0.9%) and examined in a LEICA EZ4 stereomicroscope. The helminths found were cleaned in saline solution, killed in heated 70% ethanol and preserved in the same solution at room temperature. For morphological and morphometric analysis, the nematodes were hydrated in distilled water, cleared in Aman's lactophenol 20%, mounted on temporary slides and examined under an Olympus BX41 microscope (Olympus, Tokyo, Japan) coupled with a drawing tube (without zoom adjustment). The illustrations were prepared in CorelDraw 2018 software and processed using Adobe Photoshop Version 21.0.2 software.

The measurements are presented as the values of the holotype followed by the mean of the paratypes and range in parentheses (reported in micrometres unless otherwise indicated); the metrics of nematodes obtained from different host species are given in Table 1. The prevalence and mean intensity are according to Bush *et al*. (1997) and Reiczigel *et al*. (2019). The classification of amphibian hosts follows that of Segalla *et al*. (2021) and Frost (2022). We deposited the type series in the Helminthological Collection of Oswaldo Cruz Institute (CHIOC), Brazil.

**Table 1.** Morphometric data of *Cosmocercoides amapari* n. sp. from *Boana boans*, *Boana dentei* and *Boana multifasciata*

Host	*B. boans*		*B. dentei*^a^		*B. multifasciata*		Entire sample	
Characters	Males (*n* = 6)	Females (*n* = 10)	Males (*n* = 4)	Females (*n* = 6)	Males (*n* = 7)	Females (*n* = 10)	Males (*n* = 17)	Females (*n* = 26)
Total length (mm)	3.5 (3.1–3.8)	8 (7.3–9.3)	2.6 (2.3–2.8)	9 (7.5–10.4)	2.8 (2.6–3.2)	10.1 (7.2–11.5)	3 ± 0.44	9.2 ± 1.8
Greatest width	286 (227–352)	335 (216–443)	202 (157–253)	294 (224–320)	224 (160–307)	270 (211–325)	241 ± 61	301 ± 37
Body width at oesophago–intestinal junction	195 (155–240)	224 (176–272)	142 (131–160)	209 (179–237)	164 (123–205)	189 (165–224)	170 ± 38	207 ± 20
Oesophagus length	464 (439–478)	578 (560–655)	409 (370–430)	623 (580–672)	446 (400–501)	648 (568–706)	445 ± 33	620 ± 46
Oesophagus length in % of body length	13.4 (12.2–15.4)	7.3 (6–8.4)	16.1 (14.5–18.7)	7 (6.1–7.7)	15.9 (15.3–16.7)	6.6 (5–7.9)	15 ± 1.71	6.9 ± 1.1
Pharynx length	40 (37–45)	50 (43–72)	35 (29–40)	53 (45–59)	40 (32–45)	57 (51–67)	39 ± 4.4	54 ± 5.7
Pharynx width	31 (19–40)	51 (40–53)	27 (24–29)	41 (35–45)	29 (27–35)	50 (45–59)	30 ± 4.9	47 ± 4.3
Corpus length	296 (269–309)	364 (344–413)	262 (237–283)	420 (387–456)	277 (237–320)	416 (341–453)	280 ± 23	399 ± 34
Corpus width	44 (35–48)	59 (53–67)	34 (27–40)	51 (43–56)	36 (32–43)	56 (48–69)	37 ± 6.8	56 ± 6.7
Isthmus length	37 (32–45)	52 (37–61)	35 (32–37)	45 (37–53)	45 (37–56)	64 (53–69)	41 ± 7.3	55 ± 6
Isthmus width	28 (19–32)	52 (43–64)	27 (19–32)	44 (37–51)	35 (29–40)	56 (51–66)	31 ± 5.6	52 ± 4.7
Bulb length	92 (80–107)	116 (104–128)	76 (67–83)	111 (99–120)	85 (80–96)	113 (85–141)	85 ± 9.5	113 ± 13.8
Bulb width	87 (77–112)	120 (109–133)	71 (56–80)	113 (104–120)	78 (67–88)	110 (96–130)	78 ± 17.1	115 ± 8.8
Nerve ring^b^	217 (189–237)	243 (227–280)	182 (155–203)	240 (205–256)	216 (192–245)	266 (232–307)	209 ± 23.7	253 ± 26
Nerve ring in % of body length	6.3 (5.3–7.3)	3 (2.6–3.8)	7.2 (6.2–8.8)	2.7 (2.2–3.2)	7.7 (6.9–8.3)	2.7 (1.9–3.5)	7 ± 0.98	2.8 ± 0.55
Excretory pore^b^	341 (307–376)	426 (387–467)	285 (253–309)	447 (427–512)	303 (261–333)	433 (368–500)	312 ± 33.4	433 ± 41
Excretory pore in % of body length	9.8 (9–10.5)	5.2 (4.3–6.2)	11.2 (10.1–13.4)	5 (4.2–5.7)	10.8 (10–12)	4.4 (3.3–5.5)	10.5 ± 1	4.8 ± 0.64
Tail length	221 (208–249)	444 (413–499)	188 (169–197)	587 (472–720)	220 (178–247)	657 (533–895)	213 ± 22.4	562 ± 110
Tail length in % of body length	6.4 (5.8–7.1)	5.5 (4.6–6.3)	7.4 (6.5–8.5)	6.5 (5.7–7.9)	7.8 (6.8–8.9)	6.6 (5.6–7.5)	7.2 ± 0.95	6.1 ± 0.79
Spicules	135 (125–144)	–	121 (114–126)	–	140 (130–167)	–	133 ± 12.9	–
Spicules in % of body length	3.9 (3.5–4.2)	–	4.8 (4–5.4)	–	5 (4.5–6.2)	–	4.5 ± 0.7	–
Vulva^b^ (mm)	–	3.6 (3.5–4.2)	–	3.7 (3.3–4)	–	3.8 (2.9–4.5)	–	3.7 ± 0.64
Vulva in % of body length	–	45 (43–48)	–	41 (38–48)	–	38 (34–40.1)	–	40.3 ± 12.3
Egg length	–	75 (65–81)	–	82 (75–86)	–	71 (63–78)	–	75 ± 5.2
Egg width	–	40 (33–51)	–	48 (43–52)	–	43 (37–47)	–	43 ± 3.3

All measurements are in micrometres unless otherwise indicated.

a Type series.

b From anterior end.

Some specimens were post-fixed in 1% osmium tetroxide, dehydrated in an increasing ethanol series and critical point dried in carbon dioxide. The nematodes were mounted on metallic stubs, coated with gold palladium and examined under a Vega3 (TESCAN, Brno, Czech Republic) scanning electron microscope in the Laboratory of Structural Biology, Biological Sciences Institute, Federal University of Pará (UFPA), Brazil.

### Molecular analyses and phylogenetic study

For molecular analyses, 1 male specimen was transferred to a microtube with 100% ethanol and stored in a freezer at −20°C; the anterior and posterior portions were cut and analysed by light microscopy for morphological identification of the analysed sample. Genomic DNA was extracted using a Chelex Molecular Biology Grade Resin Kit, according to the manufacturer's instructions. The partial fragment of the mitochondrial cytochrome c oxidase subunit 1 (*cox*1) was amplified by polymerase chain reaction (PCR), using specific primers and cycles condition following the protocols established by Chen *et al*. (2018*a*). The PCR products were visualized on a 1% agarose gel to determine the yield and size of the amplified fragments and were purified using a QIAquick PCR Purification kit. The amplicons’ sequence reaction followed the protocol of the Big Dye^®^ Terminator v.3.1 Cycle Sequencing kit, and were sequenced in a DNA ABI 3730 DNA Analyzer at the Human Genome Stem Cell Research Center, Biosciences Institute, University of São Paulo (USP), Brazil.

For phylogenetic analyses, the obtained sequences were edited using Geneious 7.1.3 software (Kearse *et al*., 2012) and compared (using BLAST algorithm) with the data deposited in the National Center for Biotechnology Information (NCBI) (htttp://www.ncbi.nml.nih.gov). The sequences were aligned and trimmed using Muscle (Edgar, 2004) in Geneious 7.1.3 software (Kearse *et al*., 2012). The stop codons were verified according to the translation frame and parameter for invertebrate mitochondrial DNA (translation frame 1, invertebrate mitochondrial), using Geneious 7.1.3 (Kearse *et al*., 2012). Regions poorly aligned and characters with gaps in any sequences were excluded from subsequent analyses (Tran *et al*., 2015).

The phylogenetic trees were performed with maximum-likelihood (ML) using RAxML (Guindon and Gascuel, 2003) and the analyses were carried out in CIPRES Science Gateway (Miller *et al*., 2010). ML inference was implemented using bootstrap support values of 1000 repetitions, and only nodes with bootstrap values greater than 70% were considered well-supported. The trees were edited using FigTree v1.3.1 software (Rambaut, 2009). We used *Falcaustra* sp. and *Falcaustra sinensis* Liu *et al*., 2011 (Nematoda: Kathlaniidae) as an outgroup (access numbers: MN729572 and MF113223, respectively).

## Results

### Systematics

Family: Cosmocercidae Travassos, 1925

Genus: *Cosmocercoides* Wilkie, 1930

Species: *Cosmocercoides amapari* n. sp. Rebêlo, Santos and Melo , 2022

### Taxonomic summary

*Type host: Boana dentei* (Bokermann, 1967) (Amphibia: Hylidae: Hylinae).

*Additional hosts: Boana boans* (Linnaeus, 1758) and *Boana multifasciata* (Günther, 1859) (Amphibia: Hylidae: Hylinae).

*Type locality:* Cancão Municipal Natural Park, Serra do Navio municipality, Amapá, Brazil (0°54′8.68″N, 52°0′19.62″W).

*Site of infection:* Large intestine.

*Infection parameters: Boana dentei*, prevalence 11.1% (3 infected hosts out of 27), mean intensity 5 (1–8); *Boana boans*, prevalence 10.71% (3 infected hosts out of 28), mean intensity 11.7 (3–18); *Boana multifasciata*, prevalence 19.61% (10 infected hosts out of 51), mean intensity 4.11 (1–11).

*Type material:* Holotype, male (CHIOC 39303a); allotype, female (CHIOC 39303b) and paratypes, 3 males (CHIOC 39303c), 5 females (CHIOC 39303d) were deposited in the Helminthological Collection of Oswaldo Cruz Institute.

*Additional material:* Vouchers for 13 males (CHIOC 39304–39305a) and 20 females (CHIOC 39304–39305b) were deposited in the Helminthological Collection of Oswaldo Cruz Institute.

*GenBank accession number:* OQ288108

*ZooBank registration:* urn:lsid:zoobank.org:pub:7823C8CE-89D7-4BC2-B74F-A6AAC3D54D2E

*Etymology:* The new species is named after the Amapari river that rises in the municipality of Serra do Navio and bathes Amapá.

**Fig. 1.**
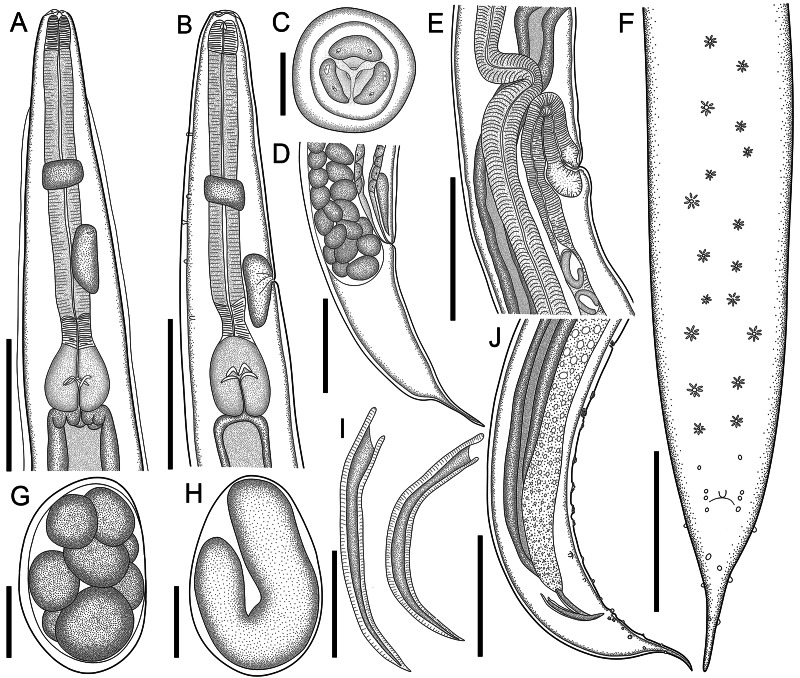
Line drawings of *C. amapari* n. sp. from Brazilian Amazon. (A) Anterior end of male, lateral view; (B) anterior end of female, lateral view; (C) anterior end of male, apical view; (D) posterior end of female, lateral view; (E) vulva region, lateral view; (F) posterior end of male, ventral view; (G) egg in morula stage; (H) embryonated egg in the uterus; (I) spicules; (J) posterior end of male, lateral view. Scale bars: A = 150 *μ*m; B, D, E, F, J = 200 *μ*m; C, G, H = 25 *μ*m; I = 100 *μ*m.

**Fig. 2.**
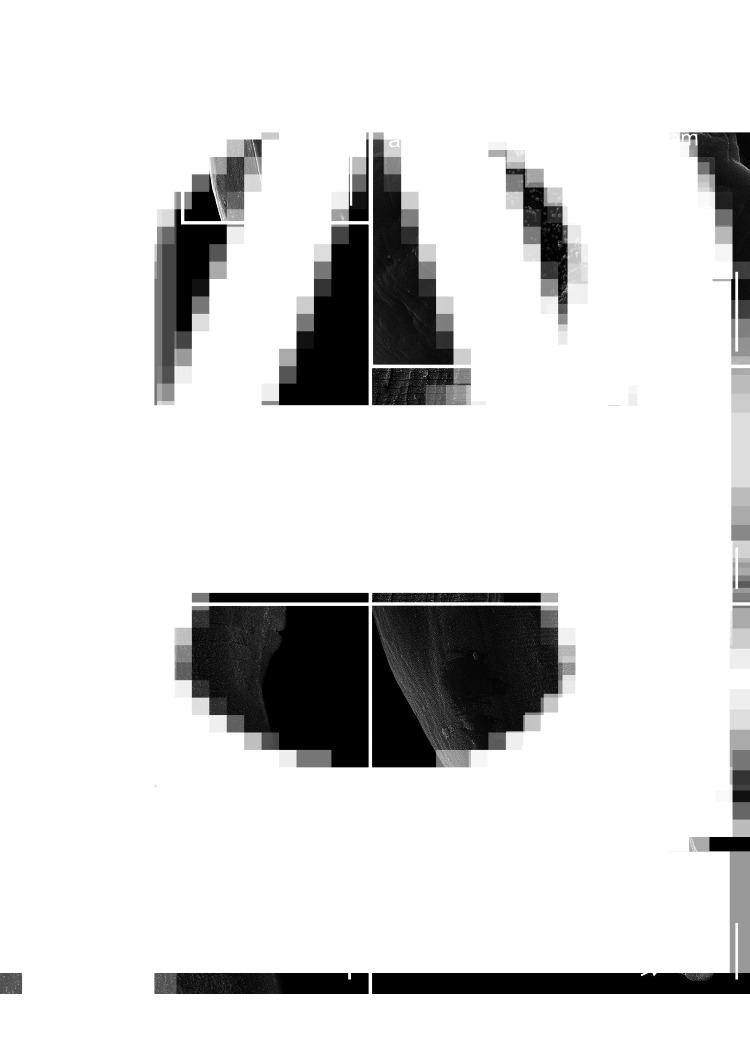
Scanning electron micrographs of *Cosmocercoides amapari* n. sp. from Brazilian Amazon, males. (A) Entire body; inset: details of lateral alae; (B) anterior end, apical view (arrows: am – amphidial pores; asterisk: sp – papillae); (C) excretory pore; (D) disposition of adcloacal papillae (arrows: acp – adcloacal papillae; up – large unpaired papilla); (E) details of rosette papillae (line: ep – external punctations; ip – internal punctations); (F) disposition of postcloacal papillae (arrows: pcp – postcloacal papillae). Scale bars: A = 500 *μ*m; B = 5 *μ*m; C = 20 *μ*m; D = 10 *μ*m; E, F = 20 *μ*m; inset: 100 *μ*m.

**Fig. 3.**
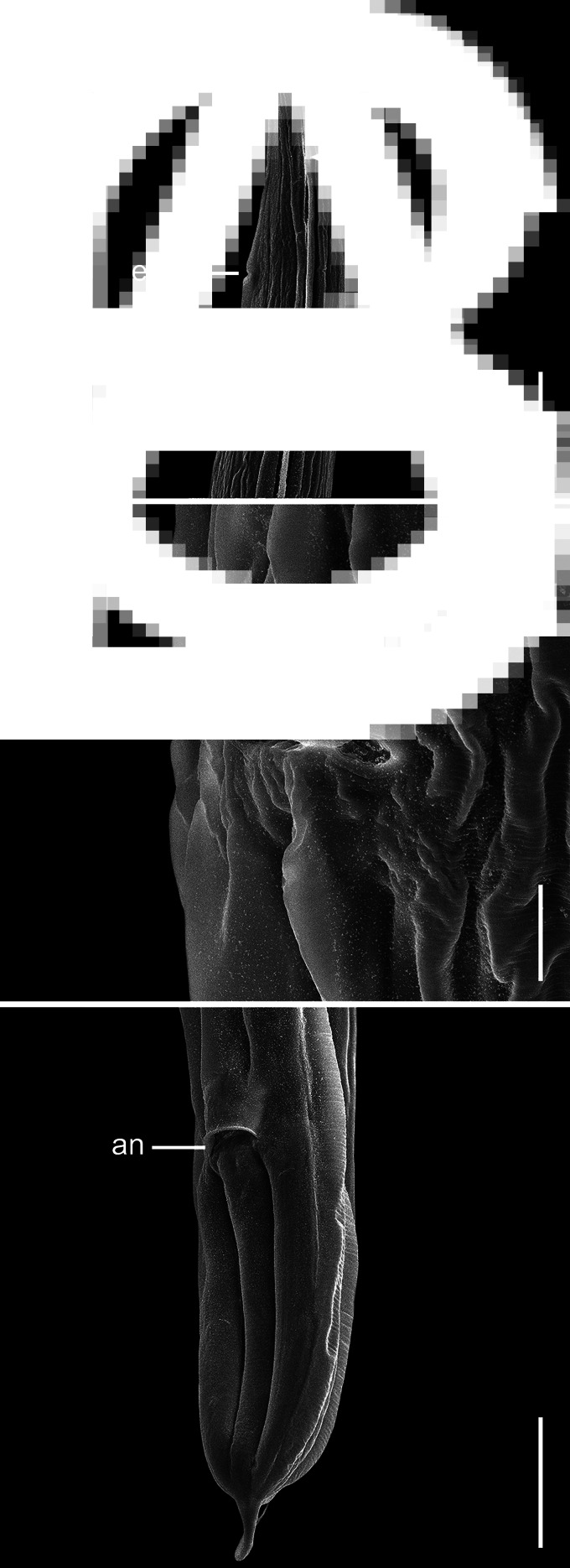
Scanning electron micrographs of *C. amapari* n. sp. from Brazilian Amazon, females. (A) Anterior end (arrows: lateral alae; line: exp – excretory pore); (B) vulva region; (C) posterior end (line: an – anus). Scale bars: A = 100 *μ*m; B = 20 *μ*m; C = 100 *μ*m.

*General.* Small and cylindrical nematodes (Fig. 1A). Cuticle with fine transverse striations. Somatic papillae present (Fig. 2A and B). Sexual dimorphism evident, females larger than males. Lateral alae present in both sexes, extending near the nerve ring region to anus in females and at the level of precloacal rosette papillae in males (Figs 1A and 3A–C). Oral opening triangular, surrounded by 3 distinct lips: dorsal lip with 2 sessile papillae, 2 subventral lips with 1 ventral sessile papilla and lateral amphidial pores (Figs 2C and 1B). Oesophagus divided into pharynx, cylindrical corpus, small isthmus and well-developed oesophageal bulb with evident valvular apparatus (Fig. 2A and B). Nerve ring situated in the middle of oesophageal corpus (Fig. 2A and B). Excretory pore anterior to isthmus (Figs 2A and B and 1C). Tail conical, sharply pointed in both sexes (Figs 2D, F–J; 1F and 3C).

*Males* (based on the holotype and 3 paratypes, all mature specimens). Total length 2.6; 2.5 (2.3–2.8) mm. Maximum width 197; 203 (157–253). Body width at oesophago–intestinal junction 136; 143 (131–160). Oesophagus 430; 402 (370–430) in total length, corresponding to 16.5; 16 (14.5–18.7)% of the body length; pharynx 35; 35 (29–40) × 24; 28 (27–29); corpus 283; 255 (237–275) × 35; 33 (27–40); isthmus 37; 35 (32–37) × 29; 27 (19–32); bulb 75; 77 (67–83) × 72; 71 (56–80). Nerve ring located at 189; 180 (155–203) from anterior extremity, corresponding to 7.3; 7.2 (6.2–8.8)% of the body length; excretory pore located at 280; 286 (253–309) from anterior extremity, corresponding to 10.8; 11.4 (10.1–13.4)% of the body length. Spicules short, equal, slightly curved ventrally, proximal ends expanded, distal ends sharply pointed 119; 122 (114–126) long, corresponding to 4.6; 4.9 (4–5.4)% of the body length (Fig. 2I). Gubernaculum absent. Caudal papillae arranged as follows: 9–10 pairs of ventral precloacal rosette papillae, distributed in 2 longitudinal rows, paired irregularly; 1 pair of precloacal simple papilla; 2 pairs of paracloacal simple papillae and 1 unpaired papilla at anterior cloacal lip; 5 pairs of postcloacal simple papillae (first and third pairs ventral, second, fourth and fifth ventrolateral) (Fig. 2F–J and 1E and F). Each rosette composed of 2 complete rings of about 13–15 punctations around central papilla (Fig. 1E). Somatic papillae present in subdorsal rows along the body. Testis single, tubular, flexing posteriorly at last third of the body length. Tail conical 169; 194 (188–197) long, corresponding to 6.5; 7.7 (6.7–8.6)% of the body length.

*Females* (based on the allotype and 5 paratypes, all gravid specimens). Total length 7.5; 9.3 (8.3–10.4) mm. Maximum width 317; 290 (224–320). Body width at oesophago–intestinal 237; 204 (179–232). Oesophagus 615; 630 (580–672) in total length, corresponding to 8.2; 6.8 (6.1–7.7)% of the body length; pharynx 45; 54 (51–59) × 43; 40 (35–45) long; corpus 400; 424 (387–456) × 53; 51 (43–56) long; isthmus 53; 43 (37–51) × 51; 443 (37–45) long; bulb 117; 109 (99–120) × 120; 111 (104–120). Nerve ring located at 237; 240 (205–256) from anterior extremity, corresponding to 3.2; 2.6 (2.2–3.1)% of the body length; excretory pore located at 427; 451 (432–512) from anterior extremity, corresponding to 5.7; 4.9 (4.2–5.4)% of the body length. Vulva slightly pre-equatorial 3.4; 3.8 (3.3–4) mm from anterior extremity, corresponding to 45; 41 (38–48)% of the body length (Figs 2E and 3B). Amphidelphic genital system formed by an anterior muscular vagina directed anteriorly in proximal half; flexed posteriorly in distal portion, divided into 1 anterior and a posterior uterus (Fig. 2E). Ovary directed anteriorly to vagina not extending beyond bulb. Uteri, containing numerous eggs in different stages of development, embryonated eggs and free-stage larvae close to vulva (Fig. 2G and H). Eggs size 75; 83 (82–86) × 45; 48 (43–52) (based on 10 embryonated eggs). Tail conical 595; 586 (472–720) long, corresponding to 7.9; 6.3 (5.7–7.1)% of the body length.

*Variability:* Values of body length and related metrical features in specimens from *B. boans* were somewhat higher than those of the specimens obtained from *B. dentei* and *B. multifasciata*, although most of the metric features overlapped (Table 1). The pattern of caudal papillae did not vary among the samples of the 3 hosts analysed.

### Remarks

The new species belongs to the genus *Cosmocercoides* based on molecular data and the general morphology of males, as the presence of papillae surrounded by punctations (rosette papillae) and not ornamented with sclerotized supports (plectanes) on their caudal region. The main morphological characteristics used to distinguish species of the genus are the pattern of caudal papillae; size and shape of spicules and gubernaculum (if present), length of tail and the presence or absence of lateral alae and somatic papillae (Draghi *et al*., 2020; Dos Anjos *et al*., 2021).

The 28 species reported for the genus *Cosmocercoides* can be divided into 2 groups based on the presence and absence of gubernaculum. The first group includes 23 species in which the gubernaculum is present: *Cosmocercoides tibetanum* Baylis, 1927; *C. dukae* Holl, 1928; *C. pulcher* Wilkie, 1930; *Cosmocercoides variabilis* Harwood, 1930; *Cosmocercoides skrajabini* Ivanitzky, 1940; *Cosmocercoides bufonis* Karve, 1944; *Cosmocercoides multipapillata* Khera, 1958; *Cosmocercoides rickae* Ogden, 1966; *Cosmocercoides nainitalensis* Arya, 1979; *Cosmocercoides barodensis* Rao, 1979; *Cosmocercoides lanceolatus* Rao, 1979; *Cosmocercoides oligodentis* Wang *et al*., 1981; *Cosmocercoides ranae* Wang *et al*., 1981; *Cosmocercoides speleomantis* Ricci, 1987; *Cosmocercoides tridens* Hasegawa, 1989; *Cosmocercoides karnatacaensis* Rizvi, 2009; *C. sauria* Ávila *et al*., 2010; *Cosmocercoides kiliwai* Martínez-Salazar *et al*., 2013; *Cosmocercoides himalayanus* Rizvi and Bursey, 2014; *Cosmocercoides malayensis* Bursey *et al*., 2015; *C. tonkinensis* Tran *et al*., 2015; *C. qingtianensis* Chen *et al*., 2018 and *C. wuyiensis* Liu *et al*., 2019.

Of the above-mentioned species, the number of caudal papillae in the new species is similar to the following: *C. bufonis*, *C. ranae* and *C. wuyiensis*. However, the new species has a pattern of rosette papillae of 18–20:0:0 and a pattern of simple papillae of 2:4:10 *vs C. bufonis* 18–26:2:6 and simple papillae of 0:0:20. Additionally, the new species differ by having smaller spicules compared to *C. bufonis* (114–126 in *C. amapari* n. sp. *vs* 190–260 in *C. bufonis*), and by the presence of somatic papillae (absent in *C. bufonis*). The new taxon can also be easily distinguished from *C. ranae* by the pattern of rosette papillae (18–20:0:0 in *C. amapari vs* 20:0:0 in *C. ranae*) and simple caudal papillae (2:4:10 in *C. amapari vs* 8:0:8 in *C. ranae*). Moreover, they also differ by the length of the spicules, 114–126 in the new species and 192 in *C. ranae* and by the presence of somatic papillae (absent in *C. ranae*).

*Cosmocercoides wuyiensis* differs from the new species by the number and pattern of rosette and simple papilla (18–20:0:0 rosette papillae and 2:4:10 simple papillae in *C. wuyiensis*). They also differ by length of the spicules, in *C. amapari* the spicules are equal in length, measuring 114–126 while in *C. wuyiensis* the spicules are unequal in length and width (151–163 the smallest and 189–206 the widest); additionally, the gubernaculum is absent in the new species and present in *C. wuyiensis*.

The new species belongs to the group of species with gubernaculum absent: *Cosmocercoides microhylae* Wan *et al*., 1978 from Paleoartic; *Cosmocercoides kumaoni* Arya, 1991 from Oriental region and *C. lilloi* Ramallo *et al*., 2007; *C. latrans* Draghi *et al*., 2020 and *C. meridionalis* Anjos *et al*., 2021 from Neotropics.

The new species differs from *C. microhylae*, by the absence of somatic papillae along the body (present in *C. amapari* n. sp.), a different pattern of simple caudal papillae (20:2:8 in *C. microhylae vs* 2:4:10 in *C. amapari* n. sp.); the males are smaller in *C. microhylae* (2.2 mm total length *vs* 2.3–3.8 mm in *C. amapari* n. sp.); also differ in the length of the spicules, smaller in *C. amapari* n. sp. (114–126) and larger in *C. microhylae* (140) and by having a shorter tail (157 in *C. microhylae vs* 169–249 in *C. amapari* n. sp.).

*Cosmocercoides amapari* n. sp. can be distinguished from *C. kumaoni* (Oriental species) by the presence of a singular hook-shaped structure near the precloacal region (absent in *C. amapari* n. sp.); the absence of simple caudal papillae on the tail of males (present in *C. amapari* n. sp.), a different pattern of rosette papillae (24:2:10 in *C. kumaoni vs* 18–20:0:0 in *C. amapari* n. sp.) and shorter tail (130–150 in *C. kumaoni vs* 169–249 in *C. amapari* n. sp.).

Among Neotropical species, the new species differs from *C. lilloi* by the presence of postcloacal rosette papillae (absent in *C. amapari* n. sp.); and the absence of simple caudal papillae, unpaired papilla at the anterior cloacal lip, lateral alae and somatic papillae (all these morphological traits were observed in *C. amapari* n. sp.). When compared to *C. latrans*, *C. amapari* n. sp. presents a higher number of precloacal rosette papillae (6–8 in *C. latrans vs* 18–20 in *C. amapari* n. sp.) and simple caudal papillae (0:2:8 in *C. latrans vs* 2:4:10 in *C. amapari* n. sp.).

The new species resembles *C*. *meridionalis* in some metric characters, such as body size, the distance of the nerve ring from the anterior end and the spicules length. However, *C. meridionalis* has a longer oesophagus (520–650 in *C. meridionalis vs* 370–501 in *C. amapari* n. sp.), different position of the excretory pore (426–571 in *C. meridionalis vs* 253–376 in *C. amapari* n. sp. from anterior extremity) and longer tail (290–446 in *C. meridionalis vs* 169–249 in *C. amapari* n. sp.). In addition, *C. meridionalis* has different pattern and distribution of rosette papillae (22:2:2 in *C. meridionalis vs* 18–20:0:0 in *C. amapari* n. sp.), with paracloacal and postcloacal rosette papillae (absent in *C. amapari* n. sp.) and a small number of simple caudal papillae on the tail (0:0:6 in *C. meridionalis vs* 2:4:10 in *C. amapari* n. sp.).

*Cosmocercoides sauria* can be easily distinguished from *C. amapari* by the presence of gubernaculum in former species. Additionally, *C. sauria* also differs from *C. amapari* n. sp. in body length (1.3 in *C. sauria vs* 2.3–3.8 in *C. amapari* n. sp.), by having shorter spicules (104 in *C. sauria vs* 114–167 in *C. amapari* n. sp.), shorter tail (54 in *C. sauria vs* 169–249 in *C. amapari* n. sp.), absence of somatic papillae (present in *C. amapari* n. sp.), smaller number of precloacal rosette papillae (8 in *C. sauria vs* 18–20 in *C. amapari* n. sp.) and simple caudal papillae (0:1:4 in *C. sauria vs* 2:4:10 in *C. amapari* n. sp.).

### Molecular analyses and phylogenetic study

The mitochondrial *cox*1 sequence obtained from *C. amapari* n. sp. has 417 bp in length. Our search for similar sequences deposited in GenBank resulted in 3 other *cox1* sequences from *Cosmocercoides* (*C. pulcher* – accession no. MH178310, *C. qingtianensis* – accession no. MH178305 and *C. wuyiensis* accession no. MK956953) (Table 2). Pairwise comparison between *C. amapari* n. sp. and *C. pulcher* showed 19% nucleotide divergence, and the divergence between *C. amapari* n. sp. and *C. qingtianensis* was 19% (supplementary Table 1). We did not include *C. wuyiensis* in our analysis and comparisons due to the number of indels in the sequences.

**Table 2.** Representatives of *Cosmocercoides* spp. used for phylogenetic analyses related to information on host, locality and GenBank ID

Species	Host species	Collection site	GenBank ID	References
*Cosmocerca albopunctata* Alcantara and Silva, 2022	*Chiasmocleis albopunctata* (Boettger, 1885)	Gavião Peixoto, São Paulo, Brazil	OP153854	Alcantara *et al*. (2022)
*C. albopunctata*	*Ch. albopunctata*	Gavião Peixoto, São Paulo, Brazil	OP153856	Alcantara *et al*. (2022)
*C. amapari* n. sp.	*Boana dentei* (Bokermann, 1967)	Serra do Navio, Amapá, Brazil		Present study
*Cosmocerca japonica* Yamaguti, 1938	*Rhacophorus arboreus* (Okada & Kawano, 1924)	Niigata, Niitsu, Japan	LC052756	Sato *et al*. (2015)
*C. japonica*	*Rhacophorus schlegelii* (Günther, 1858)	Niigata, Maki, Japan	LC052757	Sato *et al*. (2015)
*C. japonica*	*Pelophylax porosus* (Cope, 1868)	Niigata, Shibata, Japan	LC052758	Sato *et al*. (2015)
*C. japonica*	*Bufo formosus* Boulenger, 1883	Niigata, Sado, Japan	LC052759	Sato *et al*. (2015)
*C. japonica*	*R. arboreus*	Niigata, Sado, Japan	LC052760	Sato *et al*. (2015)
*C. japonica*	*Hyla japonica* (Günther, 1859)	Niigata, Sado, Japan	LC052761	Sato *et al*. (2015)
*C. japonica*	*Rana ornativentris* Werner, 1903	Niigata, Sado, Japan	LC052762	Sato *et al*. (2015)
*C. japonica*	*R. ornativentris*	Niigata, Sado, Japan	LC052763	Sato *et al*. (2015)
*C. japonica*	*Lithobates catesbeianus* (Shaw, 1802)	Kanto district, Japan	LC052764	Sato *et al*. (2015)
*C. japonica*	*Cynops pyrrhogaster* (Boie, 1826)	Kanto district, Japan	LC052765	Sato *et al*. (2015)
*C. japonica*	*Cy. pyrrhogaste*r	Kanto district, Japan	LC052766	Sato *et al*. (2015)
*C. japonica*	*Fejervarya kawamurai* Tjong *et al*., 2011	Kanto district, Japan	LC052767	Sato *et al*. (2015)
*C. japonica*	*Glandirana rugosa* (Temminck and Schlegel, 1838)	Kanto district, Japan	LC052768	Sato *et al*. (2015)
*C. japonica*	*Rh. schlegelii* (Günther, 1858)	Oita, Shonai, Japan	LC052769	Sato *et al*. (2015)
*C. japonica*	*R. ornativentris* Werner, 1903	Oita, Kokonoe, Japan	LC052770	Sato *et al*. (2015)
*Cosmocerca ornata* (Dujardin, 1845) Diesing, 1861	*Sylvirana spinulosa* (Smith, 1923)	Dayaoshan, Guangxi province, China	MT108304	Chen *et al*. (2020)
*Cosmocerca parva* Travassos, 1925	*Dendropsophus nanus* (Boulenger, 1889)	Gavião Peixoto, São Paulo, Brazil	OP153855	Alcantara *et al*. (2022)
*C. parva*	*Leptodactylus podicipinus* (Cope, 1862)	Boa Esperanca do Sul, São Paulo, Brazil	OP153857	Alcantara *et al*. (2022)
*C. parva*	*L. podicipinus*	Araraquara, São Paulo, Brazil	OP153858	Alcantara *et al*. (2022)
*Cosmocerca podicipinus* Baker and Vaucher, 1984	*Pseudis platensis* Gallardo, 1961	Cocalinho, Mato Grosso, Brazil	OP153859	Alcantara *et al*. (2022)
*C. podicipinus*	*Leptodactylus latrans* (Steffen, 1815)	Cocalinho, Mato Grosso, Brazil	OP153860	Alcantara *et al*. (2022)
*C. podicipinus*	*L. latrans*	Cocalinho, Mato Grosso, Brazil	OP153861	Alcantara *et al*. (2022)
*C. podicipinus*	*Physalaemus centralis* Bokermann, 1962	Cocalinho, Mato Grosso, Brazil	OP153862	Alcantara *et al*. (2022)
*C. podicipinus*	*Leptodactylus latrans*	Cocalinho, Mato Grosso, Brazil	OP153863	Alcantara *et al*. (2022)
*C. podicipinus*	*Boana caiapó* Pinheiro *et al*., 2018	Cocalinho, Mato Grosso, Brazil	OP153864	Alcantara *et al*. (2022)
*C. podicipinus*	*B. caiapo*	Cocalinho, Mato Grosso, Brazil	OP153865	Alcantara *et al*. (2022)
*Cosmocercoides pulcher* Wilkie, 1930	*Bufo japonicus* Temminck & Schlegel, 1838	Tokyo, Japan	MH178306	Chen *et al*. (2018*a*, 2018*b*)
*C. pulcher*	*B. japonicus*	Tokyo, Japan	MH178307	Chen *et al*. (2018*a*, 2018*b*)
*C. pulcher*	*B. japonicus*	Tokyo, Japan	MH178308	Chen *et al*. (2018*a*, 2018*b*)
*C. pulcher*	*B. japonicus*	Tokyo, Japan	MH178309	Chen *et al*. (2018*a*, 2018*b*)
*C. pulcher*	*B. japonicus*	Tokyo, Japan	MH178310	Chen *et al*. (2018*a*, 2018*b*)
*Cosmocercoides qingtianensis* Chen *et al*., 2018	*Bufo gargarizans* Cantor, 1842	Jiaozuo, Henan province, China	MH178303	Chen *et al*. (2018*a*, 2018*b*)
*C. qingtianensis*	*B. gargarizans*	Jiaozuo, Henan province, China	MH178304	Chen *et al*. (2018*a*, 2018*b*)
*C. qingtianensis*	*B. gargarizans*	Jiaozuo, Henan province, China	MH178305	Chen *et al*. (2018*a*, 2018*b*)
*C. qingtianensis*	*B. gargarizans*	Jiaozuo, Henan province, China	MH032775	Chen *et al*. (2018*a*, 2018*b*)
*C. qingtianensis*	*B. gargarizans*	Jiaozuo, Henan province, China	MH032776	Chen *et al*. (2018*a*, 2018*b*)
*C. qingtianensis*	*B. gargarizans*	Jiaozuo, Henan province, China	MH032777	Chen *et al*. (2018*a*, 2018*b*)
*Cosmocerca* sp.	*Duttaphrynus melanostictus* (Schneider, 1799)	Jinghong, Yunnan province, China	MT108305	Chen *et al*. (2020)
*Cosmocerca simile* Chen *et al*., 2020	*B. gargarizans*	Yuyao, Zhejiang province China	MN833301	Chen *et al*. (2020)
*C. simile*	*B. gargarizans*	Yuyao, Zhejiang province China	MN833303	Chen *et al*. (2020)
*Falcaustra sinensis* Liu *et al*., 2011	*Indotestudo elongata* (Blyth, 1854)	China	MF113223	Li *et al*. (2018)
*Falcaustra* sp.	*Physignathus cocincinus* Cuvier, 1829	Thua Thien-Hue province, Vietnam	MN729572	Van Ha *et al*. (2021)

**Fig. 4.**
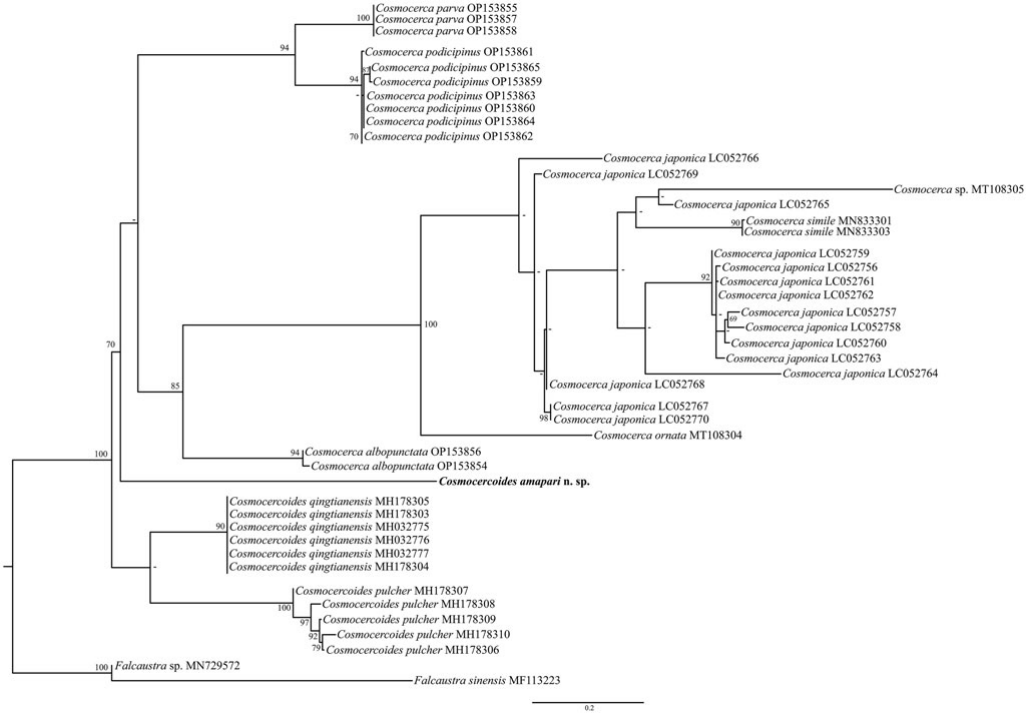
ML phylogenetic topology based on the partial *cox*1 sequence data using *Falcaustra* sp. and *Falcaustra sinensis* Liu *et al*., 2011 (Nematoda: Kathlaniidae) as outgroup indicating the position of *C. amapari* n. sp. and the phylogenetic relationships of the representatives of the Cosmocercidae. GenBank accession numbers follow each taxon. Support values are above or below nodes: bootstrap scores <70% are not shown or are represented by a dash. Branch-length scale bar indicates the number of substitutions per site.

ML phylogenetic analysis revealed that the new species sequence was related as a sister group of a large clade with all the *Cosmocerca* spp. (70% bootstrap). Sequences of *C. pulcher* and *C. qingtianensis* parasites of *Bufo japonicus formosus* Matsui, 1984 from China and *Bufo gargarizans* Cantor, 1842 from Tokyo, Japan, respectively, formed a monophyletic cluster (40% bootstrap) sister to the large clade that clustered sequences from *Cosmocerca* spp. + *C. amapari*. The clade that groups the *Cosmocerca* spp. sequences were subdivided into 2; a smaller clade that groups together sequences from *Cosmocerca parva* parasite of *Dendropsophus nanus* (Boulenger, 1889) and *Leptodactylus podicipinus* (Cope, 1862), both from Brazil and *Cosmocerca podicipinus* (94% bootstrap) parasite of *Pseudis platensis* Gallardo, 1961, *Leptodactylus luctator* (Steffen, 1815), *Physalaemus centralis* Bokermann, 1962, *Boana caiapo* Pinheiro *et al*., 2018 and a larger clade that is subdivided into a monophyletic group formed by sequences from *Cosmocerca albopunctata* (94% bootstrap) parasite of *Chiasmocleis albopunctata* (Boettger, 1885) from Brazil and a branch with a sequence from *Cosmocerca ornata* parasite of *Sylvirana spinulosa* (Smith, 1923) that is related as sister species of the clade with sequences from *Cosmocerca japonica* + *Cosmocerca simile* (100% bootstrap) parasites of multiple hosts (for *C. japonica*) from Japan and *C. simile* parasite of *Bufo gargarizans* from China (Fig. 4).

## Discussion

In our study, the new species showed variability of metrical features in the samples of the different hosts analysed. Similar results were also reported for *C. pulcher* and *C. variabilis*, where the authors found those species parasitizing a broad spectrum of hosts (Harwood, 1930; Wilkie, 1930; Vanderburgh and Anderson, 1987; Joy and Bunten, 1997; Bursey *et al*., 2007; Bursey and Brooks, 2011; Tran *et al*., 2015; Chen *et al*., 2018*a*).

The monophyly of the Cosmocercidae family has been corroborated by several studies (Tran *et al*., 2015; Sinsch *et al*., 2019; Sinsch *et al*., 2020; Ni *et al*., 2022); nonetheless, due to the low sampling of taxa, geographic limitations and few molecular data available, the phylogenetic relationships between the genera of the family are still unclear. Sinsch *et al*. (2020) and Chen *et al*. (2020) during phylogenetic studies, using the 18S and internal transcribed spacer 1 ribosomal markers, demonstrated that the genera *Cosmocerca* and *Cosmocercoides* are phylogenetically close related. However, recent studies also showed that *Cosmocerca* and *Aplectana* are phylogenetically closer (Chen *et al*., 2021; Harnoster *et al*., 2022). Additionally, the species *Cosmocerca longicauda* (Linstow, 1885), which in the analyses of Chen *et al*. (2018*b*), Sinsch *et al*. (2019) and Sinsch *et al*. (2020) are closely related to *Cosmocercoides* (based on 18S region), was not included in these works. These results reinforce that the interspecific relationships of Cosmocercidae still need to cover more species of the family.

In our analyses, after adding sequence from the new taxon, we found the same results as previous authors. Our phylogeny showed a clade formed by *C. pulcher* + *C. qingtianensis* (with low support). In phylogenetic studies using 18S and 28S from the Oriental region, the authors recovered a clade formed by sequences of the species *C. pulcher* + *C. tonkinensis* and *C. qingtianensis* or *C. pulcher* and *C. tonkinensis* (Tran *et al*., 2015; Chen *et al*., 2020, 2021; Harnoster *et al*., 2022; Ni *et al*., 2022).

Alcantara *et al*. (2022) when describing *Cosmocerca albopunctata* Alcantara and Silva, 2022, and including sequences from *Cosmocerca* spp. from the Neotropics based on the molecular marker *cox1*, also recovered *Cosmocercoides* as a monophyletic group, but with low support. However, our analysis showed that *C. amapari* n. sp. was grouped with species of *Cosmocerca* (with low support), indicating that its phylogenetic position within the group can change with the addition of new sequences from the same region or from species that have not yet been discovered.

Thus, the low support values show that the small amount of available sequences and from different biogeographic regions are factors that may be limiting the analysis. Therefore, to confirm the monophyly of the genus, it will be necessary to add new sequences from other species of *Cosmocercoides*. Additionally, the present work and the work of Alcantara *et al*. (2022) are the only studies that analysed the phylogeny of Cosmocercidae using the *cox1* marker. Thus, based on our findings and those from recent studies (Chen *et al.*, 2021; Alcantara *et al.*, 2022) we raised the hypothesis that *Cosmocercoides* spp. might have an independent diversification process in the Neotropics.

The interspecific nucleotide divergence observed in *cox1* mtDNA between *C. amapari* n. sp. and *C. qingtianensis* was 19% and that for *C. amapari* n. sp. *vs C. pulcher* varied from 19 to 23%, supporting the genetic differences between the new taxon and these species. The divergence values among species from Oriental realm were even lower (varied from 12.50 to 18.24%); however, it presents range values commonly found for Cosmocercidae (see Chen *et al*., 2018*a*; Alcantara *et al*., 2022). The high values of divergence found here may reflect the geographical distance and/or distinct morphological features, especially the gubernaculum (present in *C. qingtianensis* and *C. pulcher*, and absent in the new taxon). According to Chen *et al*. (2018*a*) and Liu *et al*. (2019), the molecular marker *cox1* is the most suitable, practical, rapid and accurate for identifying and differentiating *Cosmocercoides* species; these authors observed pieces of evidence of interspecific variation of nucleotides in this region, even when the analysed species had morphological similarity and belonged to the same biogeographic area.

Our phylogenetic analyses also indicate that *C. pulcher* and *C. podicipinus* may represent species complex; however, additional analysis should be carried out to give support to this hypothesis. The sequences of *C. japonica* are separated into 4 small clades, not well supported, which reinforces the need for taxonomic review and evaluation of the sequences deposited in the database as suggested by Alcantara *et al*. (2022).

Finally, morphological and molecular evidence based on the *cox1* region revealed that the nematode collected in *B. boans*, *B. dentei* and *B. multifasciata* represent a new species of *Cosmocercoides*. Our study also presents important results, as it is the first species of *Cosmocercoides* in Brazil and in the Neotropical region with genetic data, adds more information to Cosmocercidae and reinforces the need for further morphological and molecular studies that clarify family evolutionary relationships.

## Supplementary material

For supplementary material accompanying this paper visit https://doi.org/10.1017/S0031182022001767. click here to view supplementary material
